# A positive feedback loop between two C-type lectins originated from gene duplication and relish promotes the expression of antimicrobial peptides in *Procambarus clarkii*

**DOI:** 10.3389/fimmu.2022.1021121

**Published:** 2022-10-24

**Authors:** Xiaoling Dai, Mengling Sun, Ximei Nie, Yuqi Zhao, Hao Xu, Zhengxiao Han, Tianheng Gao, Xin Huang, Qian Ren

**Affiliations:** ^1^ Jiangsu Province Engineering Research Center for Aquatic Animals Breeding and Green Efficient Aquacultural Technology, College of Marine Science and Engineering, Nanjing Normal University, Nanjing, China; ^2^ Freshwater Fisheries Research Institute of Jiangsu Province, Nanjing, China; ^3^ Institute of Marine Biology, College of Oceanography, Hohai University, Nanjing, China

**Keywords:** C-type lectin, gene duplication, pattern recognition, relish, antimicrobial peptides, immune regulation, *Procambarus clarkii*

## Abstract

Gene duplication (GD) leads to the expansion of gene families that contributes organisms adapting to stress or environment and dealing with the infection of various pathogens. C-type lectins (CTLs) in crustaceans undergo gene expansion and participate in various immune responses. However, the functions of different CTL produced by GD are not fully characterized. In the present study, two CTL genes (designated as *PcLec-EPS* and *PcLec-QPS*, respectively) were identified from *Procambarus clarkii*. *PcLec-EPS* and *PcLec-QPS* originate from GD and the main difference between them is exon 3. PcLec-EPS and PcLec-QPS respectively contains EPS and QPS motif in their carbohydrate recognition domain. The mRNA levels of *PcLec-EPS* and *PcLec-QPS* in hemocytes, gills, intestine and lymph underwent time-dependent enhancement after D-Mannose and D-Galactose challenge. Recombinant PcLec-EPS and PcLec-QPS could bind to carbohydrates and microbes, and agglutinate bacteria. The results of experiments on recombinant protein injection and RNA interference indicate that PcLec-EPS and PcLec-QPS can respectively strong recognize and bind D-Mannose and D-Galactose, activate the Relish transcriptional factor, and further upregulate the expression of different antimicrobial peptides (AMPs). In addition, these two CTLs and Relish could positively regulate the expression of each other, suggesting that there is a positive feedback loop between two CTLs and Relish that regulates the expression of AMPs. It may contribute to the expansion of the immune response for host quickly and efficiently eliminating pathogenic microorganisms. This study provides new knowledge for clear understanding the significance and function of different CTL generated by GD in immune defenses in crustacean.

## Introduction

Gene duplication (GD) is a major source of genetic innovation. In eukaryotic organisms, the vast number of genes is in large part due to GD. In a GD event, one gene gives rise to two genes by unequal crossing over, retrotransposition, or chromosomal (or genome) duplication (1). The duplicated genes remain in the same genome and therefore are paralogues and in different genome as orthologues. GD is believed to play a vital role in evolution by providing raw material for the generation of new genes, which, in turn, facilitate the generation of new functions (1). GD is also an important process for increasing the protein diversity that allows organisms to adapt to new or different environments (2). In the immune system, GD is one of the molecular mechanisms that involved in evolution of immune molecules (3). Compare with other genes, immune genes in mammals, plants, and insects significantly evolve faster in order to adapt the ever-changing pathogenic microorganisms (4–6). The gene family expansion caused by GD contributes to deal with the infection of various pathogens from the complex environment (7, 8).

Invertebrates lack the typical adaptive immunity of vertebrates and mostly rely on the robust innate immunity to defense against infection by invading pathogens. The first step of innate immune responses is the recognition of pathogens by host pattern recognition receptors (PRRs) (9). PRRs can recognize and bind to pathogen-associated molecular patterns (PAMPs) of foreign microorganisms, such as lipopolysaccharides, peptidoglycans, lipoteichoic acid, and β-1,3-glucans (10, 11). The ‘non-self’ recognition further initiates the rapid humoral responses (clotting, melanization, production of antimicrobial peptides-AMPs) and cellular responses (encapsulation, phagocytosis, and autophagy) (12, 13). The PRRs of invertebrates are diverse, including peptidoglycan recognition proteins (PGRPs), Gram-negative bacteria-binding proteins (GNBP) or lipopolysaccharide and β-1,3-glucan binding proteins (LGBPs), C-type lectins (CTLs), galectins, thioester-containing proteins (TEPs), fibrinogen-related proteins (FREPs), scavenger receptors (SRs), Down syndrome cell adhesion molecules (DSCAMs) and Toll like receptors (TLRs) etc (10, 11). Some of these PRRs show remarkable gene expansion that may confer host increased ability to recognize diverse pathogenetic microorganisms. However, the details remain largely unknown.

As an example of gene expansion, CTLs are the largest and most diverse of the lectin families in animal with one or more characteristic carbohydrate recognition domain (CRD) that mediates ligand binding. The CRD forms a double-loop structure and the inner/long loop region participates in the calcium-mediated carbohydrate interaction (14). Moreover, the structure of CRD is mainly maintained by four conserved cysteine residues, which form two disulfide bridges. Among the four calcium-binding sites that exist in the CRD, the site 2 is involved in carbohydrate binding, which contains two remarkable amino acid motifs [EPN (Glu-Pro-Asn) or QPD (Gln-Pro-Asp), and WND (Trp-Asn-Asp)]. CTLs containing EPN or QPD motif in the CRD are characteristic of mannose-binding and galactose-binding, respectively (14, 15). Interestingly, these residues are variable that result in the generation of many new types of motifs, such as EP (D/K/Q), QP (N/T), WHD, FND, and MND etc (16). CTLs can bind a wide variety of ligands and exert various functions in the innate immunity, including promotion of phagocytosis, encapsulation, nodule formation, induction of prophenoloxidase activating system, activation of the respiratory burs, promotion of pathogen clearance, and act as opsonization molecules and antimicrobials (17, 18). Even so, there are still a lot of unknowns about the molecular mechanisms of pattern recognition and immune responses mediated by diversified CTL genes.

Compare with the roles of CTLs in the cellular immunity in insects and crustacean, the functions of CTLs in the humoral immunity are not well explained. Although a few studies have shown that CTLs in shrimp and crab could regulate the expression of AMPs by JNK or JAK/STAT signaling pathways (19–21), the possible downstream molecular signaling pathways induced by CTLs are far from complete. In addition, the knowledge about the regulation of expression of CTLs is not entirely clear. Several researches have preliminary confirmed that the transcriptions of CTLs in shrimp are regulated by NF-κB transcription factors (Relish and Dorsal) (22, 23), but more detailed study regarding how the expressions of host CTL genes are regulated under normal or infected conditions are still needed.

To clarify the significance and function of different CTL generated by GD in the innate immunity, we systematically explored the producing way, pattern recognition, immune responses, and expression regulation of two CTLs with EPS and QPS motif respectively (named *PcLec-EPS* and *PcLec-QPS*) from *Procambarus clarkii*. In detail, rapid-amplification of cDNA ends (RACE) and genome amplification were conducted to acquire full-length cDNAs and genome structures of *PcLec-EPS* and *PcLec-QPS*. The protein domain, evolution, and differences in amino acid sequence of PcLec-EPS and PcLec-QPS were analyzed. Their tissue distributions and temporal response to D-Mannose and D-Galactose challenges were examined by quantitative Real-Time PCR (RT-qPCR). Recombinant CTL proteins (rPcLec-EPS and rPcLec-QPS) were obtained to analyze their activities of sugar binding, bacterial binding, and bacterial agglutination *in vitro*. AMPs expressions regulation by mixture of recombinant CTL (rPcLec-EPS or rPcLec-QPS) and carbohydrates (D-Mannose or D-Galactose) was analyzed. RNA interference (RNAi) was used to explore the effects of *PcLec-EPS* and *PcLec-QPS* knockdown on the expressions of AMPs under normal or carbohydrate (D-Mannose or D-Galactose) challenge. RNAi was used to study the effects of *Relish* transcriptional factor knockdown on the expressions of AMPs that induced by rPcLec-EPS or rPcLec-QPS. Furthermore, the regulatory relationship of two CTLs, Relish, and AMPs in normal crayfishes was explored by RNAi.

## Materials and methods

### Experimental animals, carbohydrate challenge, and tissue collection

Healthy *P. clarkii* (approximately weight 10 g each) were purchased from an aquatic product market in Huaian, Jiangsu, China and kept in an aerated water tank filled with freshwater for 7 days before processing. Hemocytes, heart, hepatopancreas, gills, stomach, intestine, and lymph were collected from five crayfishes and quickly stored at –80°C for further RNA extraction. For hemocytes collection, the hemolymph was extracted from five crayfishes and placed in an equal volume of precooled anticoagulant solution (glucose, 1.47 g; citric acid, 0.48 g; trisodium citrate, 1.32 g; prepared in ddH_2_O and added to 100 mL, pH 7.3). The mixture was centrifuged at 4°C, 2000 rpm for 10 min to isolate hemocytes. For carbohydrate challenge, each crayfish was injected with 100 μL of D-Mannose (100 μg/mL) and D-Galactose (100 μg/mL) dissolved in ddH_2_O. At 0, 2, 6,12, and 24 h after carbohydrate injection, the hemocytes, gills, intestine, and lymph were selected from five random crayfishes for total RNA extraction.

### Total RNA extraction and cDNA synthesis

Total RNA was extracted from the collected samples using an RNApure high-purity total RNA rapid extraction kit (Spin-column, BioTeke, Beijing, China) in accordance with the manufacturer’s protocols. The quality of RNA was evaluated by 1% agarose gel electrophoresis. RNA concentration was measured by measuring the absorbance at a wavelength of 260:280 nm (OD260/OD280 = 1.8–2.0) by using Nanodrop 2000 (Thermo Fisher Scientific, USA). RNA (1 μg) was used to synthesize the first-strand cDNA using the TransScript All-in-One First-Strand cDNA Synthesis SuperMix for qPCR (One-step gDNA Removal, Transgen Biotech, Beijing, China). The obtained cDNA was kept at –20°C.

### Full-length cDNA cloning and genomic DNA sequence amplification

The 3′ - and 5′ -RACE-Ready cDNA samples used for RACE were obtain using SMARTer^®^ RACE 5′/3′ Kit (Clontech, Takara, Japan). Based on the partial CTL sequence acquired by transcriptome sequencing, specific forward (*PcLec-EPS*-F, **Table 1**) and reverse (*PcLec-EPS*-R and *PcLec-QPS*-R, **Table 1**) primers were designed to respectively acquire the 3′-end fragment of *PcLec-EPS* and the 5′ -end fragments of *PcLec-EPS* and *PcLec-QPS* by using Advantage 2 PCR Kit (Clontech, Takara, Japan) under the following conditions: five cycles at 94°C for 30 s and 72°C for 3 min; five cycles at 94°C for 30 s, 70 °C for 30 s, and 72°C for 3 min; and 25 cycles at 94°C for 30 s, 68°C for 30 s, and 72°C for 3 min. The RACE products were characterized *via* cloning and sequencing by a commercial company (Springen, Nanjing, China). The full-length cDNA sequences of *PcLec-EPS* and *PcLec-QPS* were obtained by overlapping EST sequences and 5′ and 3′ fragments. The genomic DNA sequences of *PcLec-EPS* and *PcLec-QPS* were acquired by PCR amplification, screening positive clones, and sequencing. Primers (*PcLec-EPS*-gF and *PcLec-EPS*-gR; *PcLec-QPS*-gF and *PcLec-QPS*-gR) used for genome amplification were listed in **Table 1**.

**Table 1:** Sequences of the primers used in this study.

Primers name	Primer sequences (5′-3′)
**RACE:**	
*PcLec-EPS*-F	GGAACCCCTACGCCCTGGTCTCTTATC
*PcLec-EPS*-R	CACATCTCGCAGCAGTCTTCAGCCTCC
*PcLec*-QPS-R	AGGGTGTTTGTCAGTCCACATTGCCAG
**Genome amplification:**	
*PcLec-EPS*-gF	TTCTTCGTCAGCAAGAAGAAG
*PcLec-EPS*-gR	CGCAAATATCTTTGGCGTGTT
*PcLec-QPS*-gF	CCAGCCAAGAACTCACATCTC
*PcLec-QPS*-gR	CATTAGCGTGCTGAGGTAGAA
**RNAi:**	
*PcLec-EPS*-iF	TAATACGACTCACTATAGGGTACGCCCTGGTCTCTTATCT
*PcLec-EPS*-iR	TAATACGACTCACTATAGGGCCACCTCAAGTACAGGTTCTAAA
*PcLec-QPS*-iF	TAATACGACTCACTATAGGGTCGTATCTGTACGACAGAGGAA
*PcLec-QPS*-iR	TAATACGACTCACTATAGGGCTAATTGACCCGGCAAGGAT
*PcRelish*-iF	TAATACGACTCACTATAGGGCTGTCCGTGGCAATGAAGTA
*PcRelish*-iR	TAATACGACTCACTATAGGGTTCTTCCTCCTCGTCCTCTT
*GFP*-iF	GCGTAATACGACTCACTATAGGTGGTCCCAATTCTCGTGGACC
*GFP*-iR	GCGTAATACGACTCACTATAGGCTTGAAGTTGACCTTGATGCC
**RT-qPCR:**	
*PcLec-EPS*-qF	AATGTTCGTCTGGCTTGGAG
*PcLec-EPS*-qR	CGCTGGGTTCATTCCCATT
*PcLec-QPS*-qF	TTCTACCTCAGCACGCTAATGT
*PcLec-QPS*-qR	GCTTCCTCTGTCGTACAGATAC
*PcRelish*-qF	CCGGCAGAGTTACTTCTACATC
*PcRelish*-qR	TCCAGGACAGCAACACATTC
*PcCrus2*-qF	CTCTCAAGGACTGCACAAGAA
*PcCrus2*-qR	GGTCGAGACAGGTGTCAAAG
*PcCrus3*-qF	GAGCTTCTCTGCTCCAACAT
*PcCrus3*-qR	GGCTTGCATGTGTGTTGTT
*PcCrus5*-qF	TCCAGACTGAAGTGTGCAAAG
*PcCrus5*-qR	AGGTCCAGAGACCATCGTATT
*PcALF3*-qF	GAGGAAGCTTCAGCTTGGATAA
*PcALF3*-qR	GGTCCACTCGTACAAACATTAGA
*PcALF5*-qF	CTCTCTGCTCTCACATCCAATAC
*PcALF5*-qR	TCTTGTTGTGTCCTCCTCATTT
*PcALF6*-qF	TTCAGGAAGTGGGAGCTTTAC
*PcALF6*-qR	CCTCAGTAGCCTTGTTCACTAC
*PcALF8*-qF	CGTCCCATCCCAAATCCTTATC
*PcALF8*-qR	GGGAGGGTGGAAGAGAATAAAC
*PcALF11*-qF	TCAGGGAAGGAAGGGTGTAT
*PcALF11*-qR	CCGAGAGAGTGTGTGTTTAAGG
18S rRNA-qF	ACCGATTGAATGATTTAGTGAG
18S rRNA-qR	TACGGAAACCTTGTTACGAC
**Protein expression:**	
PcLec-EPS-ex-F	GGATCCCCAGGAATTCCCAGAGATGAAGTAACTGTTGAC
PcLec-EPS-ex-R	GATGCGGCCGCTCGAGTTACATTTGATTAAACTGACACGC
PcLec-QPS-ex-F	GGATCCCCAGGAATTCCCTCAGATGAAGCAATTGTTGAC
PcLec-QPS-ex-R	GATGCGGCCGCTCGAGTTATTGCTTCAACTGACAAACAAA

### Bioinformatic analysis

Homology analysis was accomplished using the basic local alignment search tool (BLAST) at NCBI website (http://www.ncbi.nlm.nih.gov/BLAST/). The deduced amino acid sequences were obtained using the Expert Protein Analysis System (ExPASy) (https://web.expasy.org/translate/). Putative domains and motifs were predicted by the Simple Modular Architecture Research Tool (SMART) program (http://smart.embl-heidelberg.de/). The theoretical isoelectric point (pI) and molecular weight (Mw) were determined using ExPASy (http://web.expasy.org/compute_pi/). Multiple sequence alignment was carried out with DNAMAN software. An evolutionary tree was constructed using the neighbor-joining (NJ) algorithm in MEGA 7.0 software (24). Nodal support was assessed by 1000 bootstraps.

### Tissue distribution and expression pattern analysis of PcLec-EPS and PcLec-QPS

Two pairs of specific primers (*PcLec-EPS-*qF and *PcLec-EPS-*qR; *PcLec-QPS-*qF and *PcLec-QPS-*qR, **Table 1**) were designed and synthesized to examine the tissue distribution and expression patterns of *PcLec-EPS* and *PcLec-QPS* by RT-qPCR using the TransStart^®^ Top Green qPCR SuperMix Kit (TransGen Biotech, Beijing, China). The 10 μL of reaction system contains 5 μL of 2 × TransStart Top Green qPCR SuperMix, 0.4 μL (10 mM) each of the qF and qR primers, 1 μL of cDNA template, and 3.2 μL of PCR-Grade Water. Amplification was conducted at 94°C for 30 s, followed by 40 cycles of 94°C for 5 s, and 60°C for 30 s. Melting curve analysis was performed from 60°C to 95°C. 18S rRNA from *P. clarkii* was used as internal reference and was amplified from all samples with 18S rRNA-qF and 18S rRNA-qR primers (**Table 1**). All experiments were repeated three times, and the data were calculated using the 2^−ΔΔCT^ threshold cycle (CT) method (25). Statistical analysis was performed using Student’s *t*-test, and the level of significant difference was set at *p* < 0.05.

### PcLec-EPS and PcLec-QPS RNAi and detection of AMPs expression

Primers specific to *PcLec-EPS* (*PcLec-EPS-*iF and *PcLec-EPS-*iR, **Table 1**), *PcLec-QPS* (*PcLec-QPS-*iF and *PcLec-QPS-*iR, **Table 1**), and green fluorescent protein (*GFP-*iF and *GFP-*iR, **Table 1**) were designed to synthesize DNA template for the transcription of *PcLec-EPS*-dsRNA, *PcLec-QPS*-dsRNA, and *GFP*-dsRNA. The obtained DNA template was used to synthesize the double stranded RNAs (dsRNA) using the HiScribe™ T7 Quick High Yield RNA synthesis kit (Biolabs, USA) *in vitro*. The crayfishes were initially injected with 20 μg of *PcLec-EPS*-dsRNA, *PcLec-QPS*-dsRNA, or *GFP*-dsRNA (as control). After 24 h, 20 μg of *PcLec-EPS*-dsRNA, *PcLec-QPS*-dsRNA, or *GFP*-dsRNA was injected into the same prawn. At 24 h after the second dsRNA injection, the gills from five crayfishes were collected for RNA extraction and cDNA synthesis. The RNAi efficiency of *PcLec-EPS* and *PcLec-QPS* were checked using RT-qPCR. Expression levels of multiple AMPs [including crustin (*Crus*)(*2*, *3*, and *5*) and anti-lipopolysaccharide factor (*ALF*) (*3*, *5*, *6*, *8*, and *11*)] in the gills of *PcLec-EPS* RNAi and *PcLec-QPS* RNAi crayfishes were detected by RT-qPCR using primers (*PcCrus2-*qF and *PcCrus2-*qR; *PcCrus3-*qF and *PcCrus3-*qR; *PcCrus5-*qF and *PcCrus5-*qR; *PcALF3-*qF and *PcALF3-*qR; *PcALF5-*qF and *PcALF5-*qR; *PcALF6-*qF and *PcALF6-*qR; *PcALF8-*qF and *PcALF8-*qR; *PcALF11-*qF and *PcALF11-*qR; **Table 1**). Three independent experiments were performed in triplicate. Student’s *t*-test was conducted for statistical analysis, and *p* < 0.05 was considered statistically significant.

### Recombinant expression and purification of PcLec-EPS and PcLec-QPS

Two pair of primers (PcLec-EPS-ex-F and PcLec-EPS-ex-R; PcLec-QPS-ex-F and PcLec-QPS-ex-R; **Table 1**) were designed to respectively amplify cDNA fragment that encode *PcLec-EPS* and *PcLec-QPS*. The amplified cDNA fragment was inserted into the pGEX-6p-2 vector (Novagen, Germany) that was digested by restriction enzymes *EcoR* I and *Xho* I (NEB, USA). Recombinant plasmid was transformed into *Escherichia coli* BL21 (DE3) cells (TransGen Biotech, Beijing, China) for the expression of recombinant protein. rPcLec-EPS and rPcLec-QPS proteins with GST tag were purified using glutathione Sepharose 4B chromatography (GE Healthcare, USA) in accordance with the manufacturers’ protocols. Purified protein was separated using 12.5% SDS polyacrylamide gel electrophoresis (SDS-PAGE) and visualized using Coomassie brilliant blue R250. The concentration of recombinant protein was determined using Bradford protein assay kit (Jiancheng, Nanjing, China).

### Carbohydrates binding, microbial binding, and bacterial agglutination assays of rPcLec-EPS and rPcLec-QPS

Enzyme-linked immunosorbent assay (ELISA) was performed to analyze the ability of rPcLec-EPS and rPcLec-QPS to bind directly to D-Mannose and D-Galactose. D-Mannose or D-Galactose (50 µL, 100 µg/mL) was added to a 96-well microtiter plate, incubated overnight at 37°C, and heated at 60°C for 30 min. To prevent non-specific adsorption, each well was blocked with 200 µL of 1 mg/mL bovine serum albumin (BSA) in Tris-buffered saline (TBS, 150 mM NaCl, 10 mM Tris–HCl, pH 7.5) at 37°C for 2 h. After washed with TBS (200 µL/each well) four times, purified rPcLec-EPS or rPcLec-QPS with different concentrations in BSA–TBS (0.1 mg/mL) were added to the wells and incubated at room temperature for 3 h. The same concentration of GST protein was used as control. Each well was washed with 200 µL TBS for four times. Each well was incubated with 100 µL of mouse monoclonal anti-GST antibody (1: 2000 dilution in 0.1 mg/mL BSA–TBS) at 37°C for 2 h. The plate was washed as above and then incubated with peroxidase-conjugated goat anti-mouse IgG (1: 5000 dilution in 0.1 mg/mL BSA–TBS) at 37°C for 1 h. The plate was washed as above and then added 100 µL/well of 0.01% 3,3′,5,5′-tetramethylbenzidine (Sigma) to develop color. 2 M H_2_SO_4_ was added to stop the reaction. Absorbance was measured at 450 nm using a plate reader (BioTek Instruments, USA). Three biological repeats were used for each group. Data are presented as mean ± SD.

Three species of Gram-positive (*Staphylococcus aureus*, *Bacillus megaterium*, and *Bacillus subtilis*) and three species of Gram-negative (*Vibrio parahemolyticus*, *Vibrio alginolyticus*, and *Aeromonas hydrophila*) bacteria were used for microbial binding assay. In brief, 10 µg of purified rPcLec-EPS or rPcLec-QPS was incubated with microbes (approximately 2 × 10^8^ cells each) in midlogarithmic phase by gentle rotation for 1 h at 37 °C. After centrifugation at 6000 rpm for 5 min, the cells were collected, washed four times with TBS, and eluted with 5% SDS. The binding between microbes and recombinant protein was analyzed through 12.5% SDS–PAGE and detected by Western blot using mouse monoclonal anti-GST antibody (TransGen Biotech, Beijing, China). The bacterial cells used as controls were incubated with the GST protein and subjected to the same treatments. The experiment was repeated three times.

Gram-positive bacteria (*S. aureus*) and Gram-negative (*V. parahaemolyticus*) were used for bacterial agglutination assay. Bacteria were cultured overnight, harvested, washed twice with TBS, and suspended at 2 × 10^8^ cells/mL. In the presence or absence of 10 mM CaCl_2_, a microorganism-TBS solution (25 μL) was incubated with 25 μL recombinant protein-TBS suspension (rPcLec-EPS or rPcLec-QPS, 100 μg/mL) or BSA-TBS suspension (100 μg/mL, as negative control) at room temperature for 1 h. Agglutination was observed with microscopy.

### Effects of rPcLec-EPS and rPcLec-QPS on AMPs expression

Purified rPcLec-EPS or rPcLec-QPS (4 µg) was mixed with D-Mannose or D-Galactose (10 µg) and then injected into crayfishes. At 24 h after mixture injection, the gills from five crayfishes were collected for RNA extraction and cDNA synthesis. Samples from untreated crayfishes (Normal group) were collected as control. Expression levels of *Crus2*, *Crus3*, *Crus5*, *ALF3*, and *ALF6* in the gills of mixture (rPcLec-EPS plus D-Mannose or rPcLec-EPS plus D-Mannose) injected crayfishes were detected by RT-qPCR. The transcriptional levels of *Crus2*, *ALF3*, *ALF5*, *ALF8*, and *ALF11* in the gills of mixture (rPcLec-QPS plus D-Galactose or rPcLec-QPS plus D-Galactose) injected crayfishes were measured by RT-qPCR. Three independent experiments were performed in triplicate. The normalized data were subjected to statistical analysis followed by Student’s *t*-test. Significant difference was accepted at *p* < 0.05

### RNAi of PcLec-EPS and PcLec-QPS, carbohydrate challenge, and detection of AMPs expression

*Lec-EPS-*dsRNA, *Lec-QPS-*dsRNA, and *GFP-*dsRNA were synthesized as described above. Total 40 µg of each dsRNA was injected into crayfish. At 48 h after dsRNA injection, 100 µL of D-Mannose or D-Galactose (100 µg/mL) was injected into the same crayfish. At 24 h after carbohydrate injection, the gills were collected from five individuals. The expression level of *PcLec-EPS* in the gills at 24 h after D-Mannose challenge in *PcLec-EPS* RNAi crayfishes was detected by RT-qPCR. Samples from D-Mannose only and *GFP-*dsRNA plus D-Mannose groups were used as controls. RT-qPCR was also used to analyze the transcription level of *PcLec-QPS* in the gills at 24 h after D-Galactose challenge in *PcLec-QPS* RNAi crayfishes. D-Galactose only and *GFP-*dsRNA plus D-Galactose were used as controls. In addition, the mRNA expressions of *Crus2*, *Crus3*, *Crus5*, *ALF3*, and *ALF6* in the gills at 24 h after D-Mannose challenge in *PcLec-EPS* RNAi crayfishes were detected. The transcriptional levels of *Crus2*, *ALF3*, *ALF5*, *ALF8*, and *ALF11* in the gills at 24 h after D-Galactose challenge in *PcLec-QPS* RNAi crayfishes were measured. 18S rRNA was used as an internal reference gene for internal normalization, and samples were performed in triplicate. Student’s *t*-test was conducted for statistical analysis, and the significance was accepted when *p* < 0.05.

### RNAi of relish, injection of rPcLec-EPS and rPcLec-QPS, and detection of AMPs expression

A pair of specific primers (*PcRelish*-iF and *PcRelish*-iR, **Table 1**) with T7 promoter sequences was used to synthesize DNA template of *PcRelish*. The obtained DNA fragment was used to synthesize the *PcRelish*-dsRNA following the method described earlier. Approximately 40 μg of *PcRelish*-dsRNA or *GFP*-dsRNA (as control) was injected into each crayfish. The gills from five crayfishes were collected at 48 h after dsRNA injection. To detect the efficiency of RNAi, the transcriptional level of *PcRelish* in the gills of dsRNA (*PcRelish*-dsRNA and *GFP*-dsRNA)-injected crayfishes were analyzed by RT-qPCR using primers *PcRelish*-qF and *PcRelish*-qR (**Table 1**). Furthermore, at 48 h after *Relish-*dsRNA injection, the purified rPcLec-EPS or rPcLec-QPS (4 µg) was injected into each crayfish. At 24 h after recombinant protein injection, the gills from five crayfishes were collected for RNA extraction, cDNA synthesis, and RT-qPCR analysis. The mRNA expressions of *Crus2*, *Crus3*, *Crus5*, *ALF3*, and *ALF6* in the gills at 24 h after rPcLec-EPS injection in *Relish* RNAi crayfishes were detected by RT-qPCR. Samples from rPcLec-EPS only and *GFP*-dsRNA plus rPcLec-EPS groups were collected as controls. The transcriptional levels of *Crus2*, *ALF3*, *ALF5*, *ALF8*, and *ALF11* in the gills at 24 h after rPcLec-QPS injection in *Relish* RNAi crayfishes were analyzed by RT-qPCR. Samples from rPcLec-QPS only and *GFP*-dsRNA plus rPcLec-QPS groups were collected as controls. Student’s *t*-test was conducted for statistical analysis, and significant difference was accepted when *p* < 0.05.

### The regulatory relationship among two CTLs, relish, and AMPs

RNAi of *PcLec-EPS*, *PcLec-QPS*, and *PcRelish* was conducted as described above. The expression levels of *PcLec-EPS* and *PcLec-QPS* in the gills at 48 h after *Relish*-dsRNA injection were detected by RT-qPCR. The transcription level of *PcRelish* in the gills at 48 h after *Lec-EPS*-dsRNA and *Lec-QPS*-dsRNA injection was respectively detected by RT-qPCR. In addition, the mRNA expressions of multiple AMPs (including *Crus2*, *Crus3*, *Crus5*, *ALF3*, *ALF5*, *ALF6*, *ALF8*, and *ALF11*) in the gills at 48 h after *Relish*-dsRNA injection were detected by RT-qPCR. Group of *GFP*-dsRNA injection was set as control. Three independent experiments were performed in triplicate. Student’s *t*-test was conducted for statistical analysis, and *p* < 0.05 was considered statistically significant.

## Results

### Sequence characters and evolutionary analysis of PcLec-EPS and PcLec-QPS

**Figure 1:**
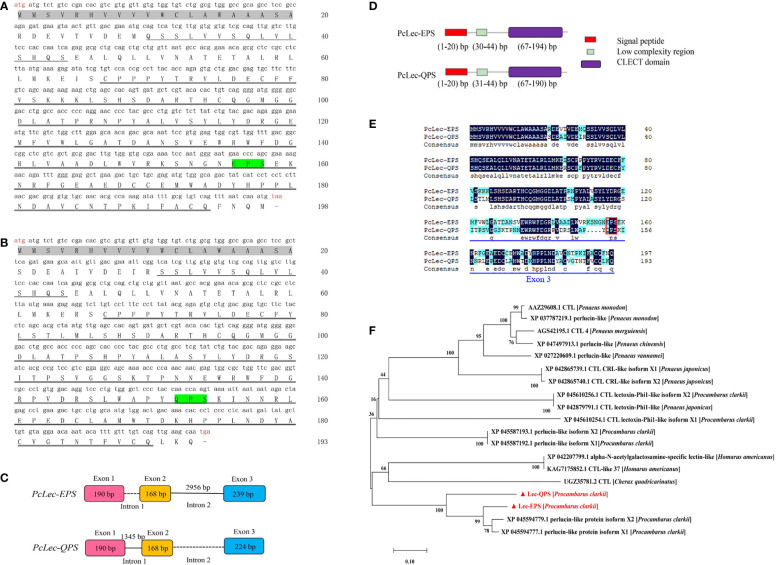
**(A)** Nucleotide sequence and deduced amino acid sequence of PcLec-EPS. **(B)** Nucleotide sequence and deduced amino acid sequence of PcLec-QPS. The start codon (atg) and stop codon (taa/tga) are marked in red. The signal peptide, low complexity region, and CLECT/CRD domain are marked with gray shadow, single underline and double underline, respectively. **(C)** Genomic structure of *PcLec-EPS* and *PcLec-QPS*. Exons, known intron, and unknown intron are represented by boxes with different color, straight line, and dotted line, respectively. The number in the boxes and above the straight line represent the number of nucleotides. **(D)** Protein structures of PcLec-EPS and PcLec-QPS. The signal peptide, low-complexity region, and CLECT/CRD domain are respectively marked in red, green, and purple boxes. The range of the nucleotide encoding the amino acids of each domain is provided. **(E)** The amino acids sequence alignment of PcLec-EPS and PcLec-QPS. The identical and similar amino acid residues were shaded in black and blue, respectively. The EPS and QPS are marked in red box. The difference in amino acids between PcLec-EPS and PcLec-QPS that underlined in blue is major caused by the difference of exon 3 sequence. **(F)** Phylogenetic analysis of PcLec-EPS, PcLec-QPS, and their homologous proteins from crustaceans was performed by MEGA 7.0. PcLec-EPS and PcLec-QPS are marked in red with triangle. The phylogenetic tree was built by the NJ algorithm, and the numbers at the nodes indicated the bootstrap value.

The full-length cDNAs of two CTLs in *P. clarkii* were obtained by RACE. The open reading frame (ORF) of *PcLec-EPS* (**Figure 1A**) and *PcLec-QPS* (**Figure 1B**) were 597 and 582 bp that encode 198 and 193 deduced amino acid (aa) residues, respectively. The genome sequences of *PcLec-EPS* and *PcLec-QPS* were obtained by PCR amplification. As shown in **Figure 1C**, the genome structure of *PcLec-EPS* and *PcLec-QPS* included three exons, one known intron and one unknown intron. The number of nucleotides that make up the exon 3 in *PcLec-EPS* was 239 bp, whereas that in *PcLec-QPS* was 224 bp. Conserved domain analysis revealed that both PcLec-EPS and PcLec-QPS contain a signal peptide (amino acids 1–20), a low complexity region (amino acids 30–44 and 31–44), and a CLECT/CRD domain (amino acids 67–194 and 67–190) (**Figure 1D**). The amino acid sequences of PcLec-EPS and PcLec-QPS were compared using DNAMAN software. The result showed that PcLec-EPS and PcLec-QPS have 69.19% of identity in the sequences of amino acids. The main difference in amino acid sequence between PcLec-EPS and PcLec-QPS is the region encoded by exon 3 (**Figure 1E**). Moreover, PcLec-EPS and PcLec-QPS respectively contains the EPS and QPS motif that specific binds to mannose and galactose. Phylogenetic analysis showed that PcLec-EPS and PcLec-QPS have a close genetic distance with two perlucin-like proteins (XP 045594779 and XP 045594777) from *P. clarkii* (**Figure 1F**). And, perlucin is also a member of the CTL family, which participates in the immune response to various stressors and defends against invading pathogens.

### Tissue distribution and expression profiles of PcLec-EPS and PcLec-QPS

**Figure 2:**
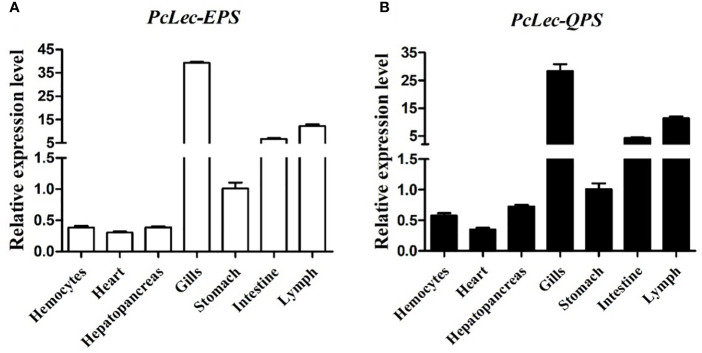
Relative expression levels of **(A)** *PcLec-EPS* and **(B)** *PcLec-QPS* in seven tissues (hemocytes, heart, hepatopancreas, gills, stomach, intestine, and lymph) were detected by RT- qPCR. 18S rRNA was used as an internal reference gene for internal normalization. The values were shown as mean ± SD, N = 5.

**Figure 3:**
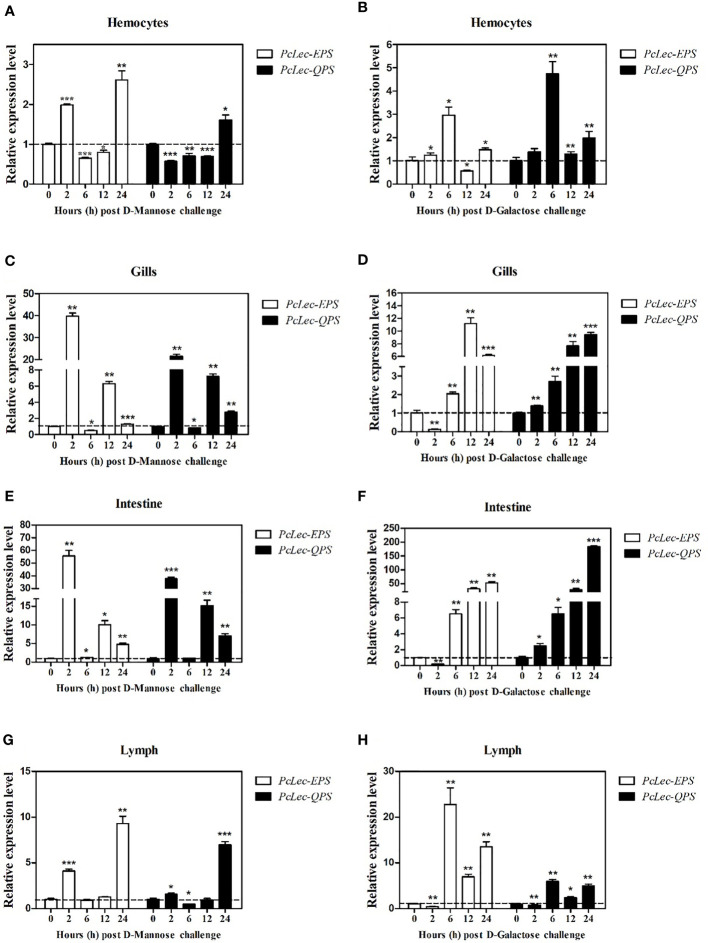
Relative transcription levels of *PcLec-EPS* and *PcLec-QPS* in the **(A, B)** hemocytes, **(C, D)** gills, **(E, F)** intestine, and **(G, H)** lymph of D-Mannose or D-Galactose challenged crayfishes. At 0, 2, 6, 12, and 24 h after D-Mannose or D-Galactose injection, the hemocytes, gills, intestine, and lymph were collected and processed for the RT-qPCR analysis of *PcLec-EPS* and *PcLec-QPS* expressions. 18S rRNA was used as an internal control. Data are presented as mean ± SD, N = 5. Asterisks indicate significant differences (*p < 0.05, **p < 0.01, and ***p < 0.001) compared to the untreated group (0 h group).

The expression levels of *PcLec-EPS* and *PcLec-QPS* in normal *P. clarkii* tissues were detected by RT-qPCR. The results showed that *PcLec-EPS* (**Figure 2A** **)** and *PcLec-QPS* (**Figure 2B** **)** are widely distributed in multiple tissues, and have higher expression levels in the gills, lymph, and intestine than that in the stomach, hepatopancreas, hemocytes, and heart. The expression patterns of *PcLec-EPS* and *PcLec-QPS* in the hemocytes, gills, intestine, and lymph challenged by D-Mannose or D-Galactose were further studied. The expression level of *PcLec-EPS* in hemocytes was greatly upregulated at 2 and 24 h after D-Mannose challenge, whereas that of *PcLec-QPS* in hemocytes was only upregulated at 24 h post D-Mannose injection (**Figure 3A** **)**. Upon D-Galactose challenge, the expression level of *PcLec-EPS* and *PcLec-QPS* in hemocytes were increased at 6 and 24 h (**Figure 3B** **)**. The mRNA expressions of *PcLec-EPS* and *PcLec-QPS* in gills were upregulated at 2, 12, and 24 h after D-Mannose challenge (**Figure 3C**) and increased from 6 to 24 h after D-Galactose challenge (**Figure 3D**). At 2, 12, and 24 h post D-Mannose injection, the expression levels of *PcLec-EPS* and *PcLec-QPS* in intestine were upregulated (**Figure 3E**). The transcriptional levels of *PcLec-EPS* and *PcLec-QPS* in intestine were increased from 6 to 24 h after D-Galactose challenge (**Figure 3F**). The mRNA expressions of *PcLec-EPS* and *PcLec-QPS* in lymph were upregulated at 2 and 24 h after D-Mannose challenge (**Figure 3G** **)**. The transcriptional levels of *PcLec-EPS* and *PcLec-QPS* in lymph were increased from 6 to 24 h after D-Galactose challenge (**Figure 3H**).

### Effects of PcLec-EPS and PcLec-QPS knockdown on the expressions of AMPs

**Figure 4:**
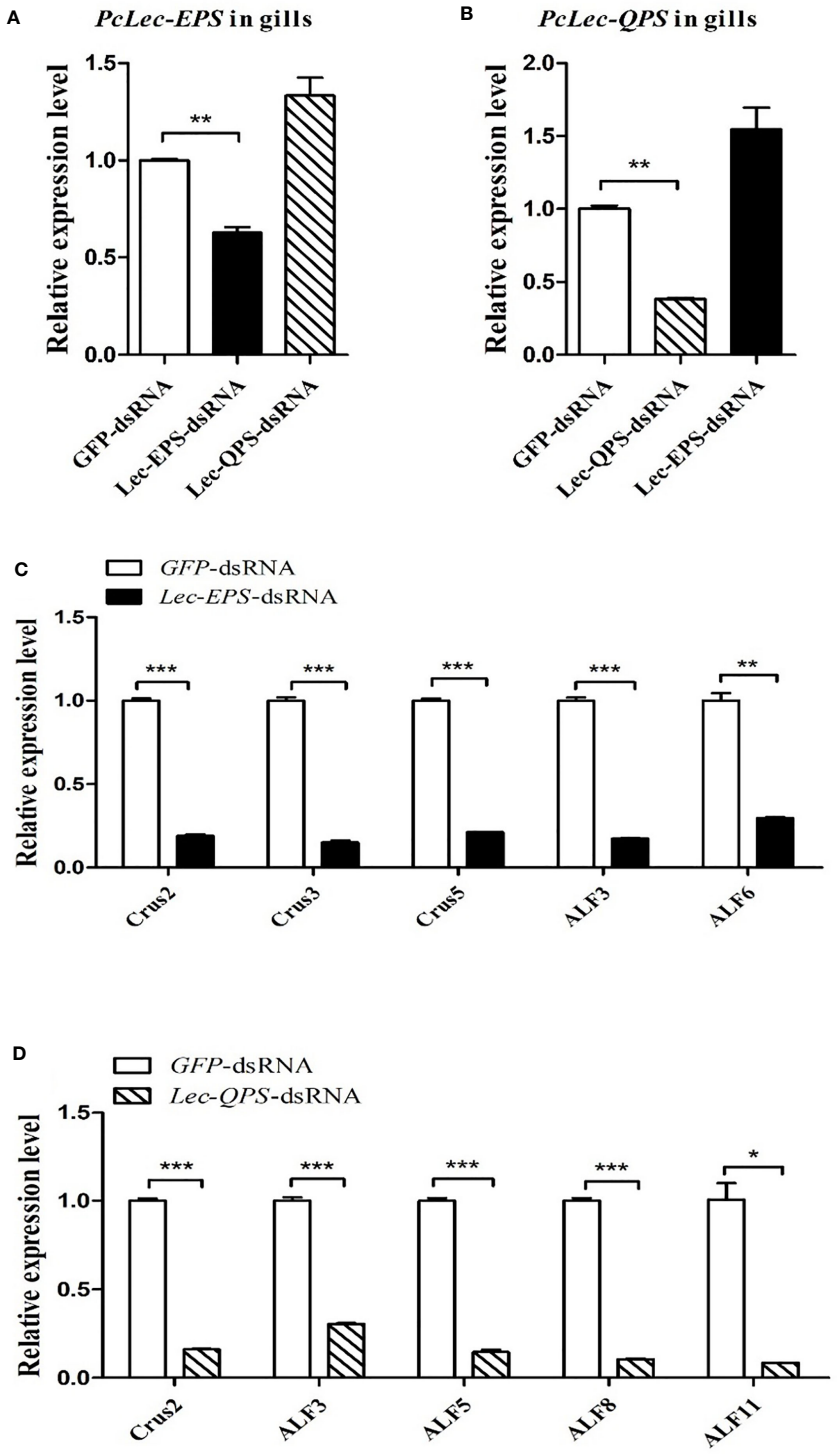
Analysis of AMPs expression levels after *PcLec-EPS* and *PcLec-QPS* RNAi. The mRNA expression levels of **(A)** *PcLec-EPS* and **(B)** *PcLec-QPS* in the gills at 48 h after dsRNA (*GFP*-dsRNA, *Lec-EPS*-dsRNA, or *PcLec-QPS-*dsRNA) injection were determined by RT-qPCR. **(C)** The transcriptional levels of *Crus2*, *Crus3*, *Crus5*, *ALF3*, and *ALF6* in the gills at 48 h after *Lec-EPS*-dsRNA injection were measured by RT-qPCR. **(D)** The expression levels of *Crus2*, *ALF3*, *ALF5*, *ALF8*, and *ALF11* in the gills at 48 h after *Lec-QPS*-dsRNA injection were analyzed by RT-qPCR. All data were normalized to *GFP*-dsRNA treated samples and 18S rRNA was used as an internal reference. Data are presented as mean ± SD, N=5. The asterisk indicated significant difference between *Lec-EPS*-dsRNA or *Lec-QPS-*dsRNA group and *GFP*-dsRNA group (*p < 0.05, **p < 0.01, and ***p < 0.001).

DsRNA-mediated RNAi was used to explore the roles of PcLec-EPS and PcLec-QPS on the production of multiple AMPs. As shown in **Figure 4A**, the expression level of *PcLec-EPS* in the gills at 48 h after *Lec-EPS*-dsRNA injection was remarkably decreased compared with the *GFP*-dsRNA injection group (as control), whereas *Lec-QPS*-dsRNA injection made no change in the expression of *PcLec-EPS*. The mRNA expression of *PcLec-QPS* in the gills of *Lec-QPS*-dsRNA-injected crayfishes was greatly decreased, whereas *Lec-EPS*-dsRNA injection made no change in the expression of *PcLec-QPS* (**Figure 4B**). These results indicated that *Lec-EPS*-dsRNA and *Lec-QPS*-dsRNA injection can specifically downregulate the expression of *PcLec-EPS* and *PcLec-QPS* in *P. clarkii*, respectively. After knockdown of *PcLec-EPS* and *PcLec-QPS*, the expression levels of multiple AMPs were detected by RT-qPCR. The results showed that mRNA expressions of *Crus2*, *Crus3*, *Crus5*, *ALF3*, and *ALF6* in the gills of *PcLec-EPS* RNAi crayfishes were significantly decreased (**Figure 4C**). Knockdown of *PcLec-QPS* could remarkably downregulate the expressions of *Crus2*, *ALF3*, *ALF5*, *ALF8*, and *ALF11* (**Figure 4D**). These results suggested that *PcLec-EPS* and *PcLec-QPS* positively regulated the expression of different AMP in *P. clarkii*.

### Carbohydrates binding, microbial binding, and bacterial agglutination activities of recombinant PcLec-EPS and PcLec-QPS proteins

**Figure 5:**
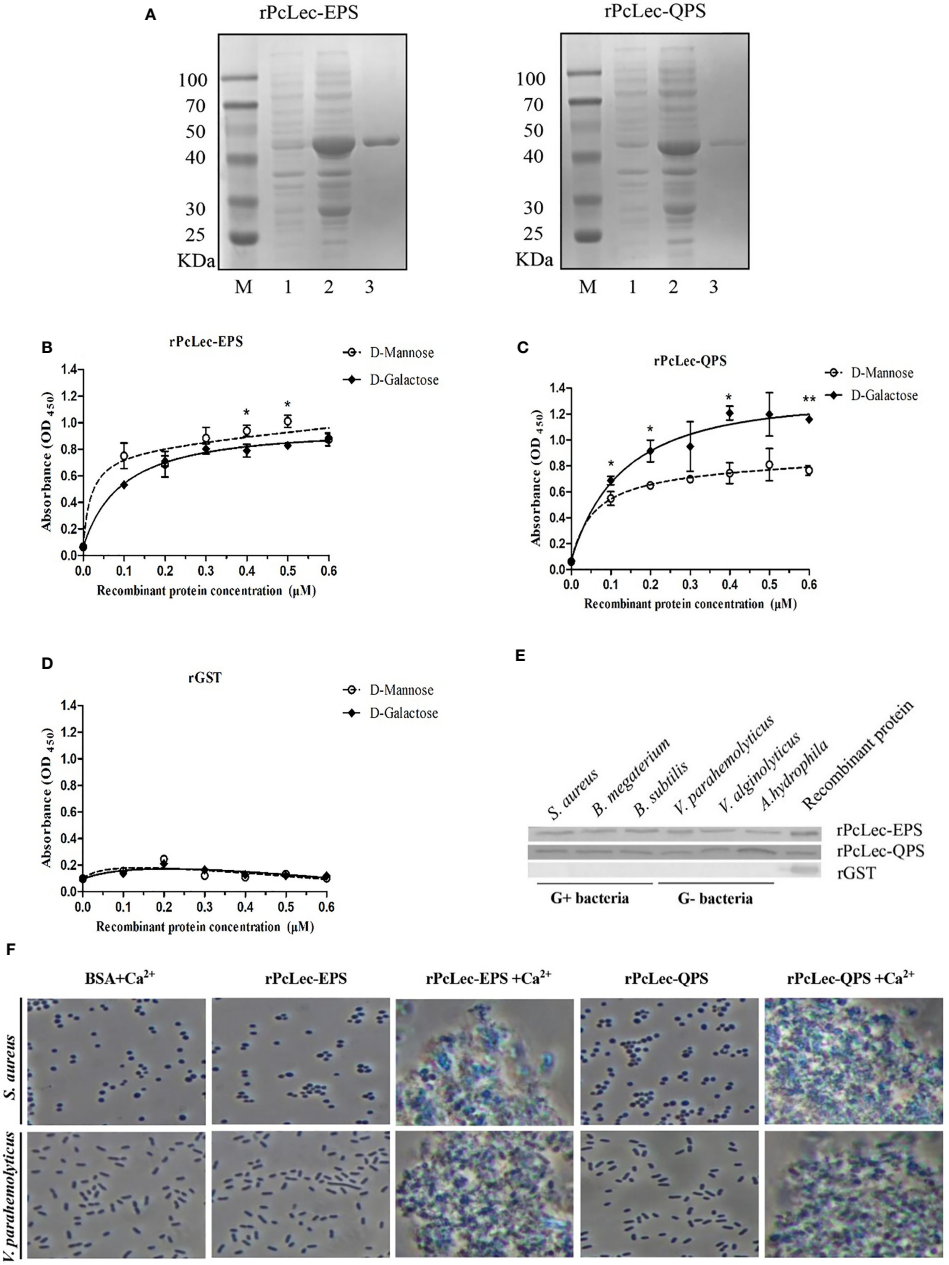
Analysis of carbohydrates binding, microbial binding, and bacterial agglutination of rPcLec-EPS and rPcLec-QPS. **(A)** Recombinant expression and purification of PcLec-EPS and PcLec-QPS. M: standard protein marker; Lane 1: total proteins of *E. coli* with recombinant plasmids without IPTG induction; Lane 2: total proteins of *E. coli* with recombinant plasmids induced with 0.5 mM IPTG; Lane 3: purified rPcLec-EPS and rPcLec-QPS. **(B)** ELISA was used to detect the binding activities of rPcLec-EPS to D-Mannose and D-Galactose. **(C)** Analysis of binding activity of rPcLec-QPS to D-Mannose and D-Galactose. **(D)** Analysis of binding activity of rGST to D-Mannose and D-Galactose. Asterisks (*p < 0.05 and **p < 0.01) indicate that there are significant differences between two different experimental groups at the same recombinant protein concentration. **(E)** Analysis of binding activity of rPcLec-EPS and rPcLec-QPS to various Gram-positive and Gram-negative bacteria. **(F)** Agglutination of S. aureus and V. parahaemolyticus by rPcLec-EPS and rPcLec-QPS. Agglutination was observed using a microscope in the presence or absence of Ca^2+^. BSA was used as a negative control.

Recombinant CTLs protein were obtained by prokaryotic expression system. PcLec-EPS and PcLec-QPS proteins were respectively estimated to have an MW of 22.4 and 21.6 kDa. The apparent molecular mass of the purified rPcLec-EPS and rPcLec-QPS were approximately 48 kDa with a GST-tag (about 26 kDa) (**Figure 5A**). ELISA assay showed that rPcLec-EPS and rPcLec-QPS can bind directly to D-Mannose and D-Galactose in a concentration-dependent manner. Moreover, rPcLec-EPS had a higher binding activity with D-Mannose than with D-Galactose, whereas rPcLec-QPS had a higher binding activity with D-Galactose than with D-Mannose (**Figures 5B, C** **)**. As a negative control, the rGST protein had no binding activity to D-Mannose or D-Galactose (**Figure 5D**). Microbial binding assay showed that both rPcLec-EPS and rPcLec-QPS can bind to all tested Gram-positive bacteria (*S. aureus*, *B. megaterium*, and *B. subtilis*) and Gram-negative bacteria (*V. parahaemolyticus*, *V. alginolyticus*, and *A. hydrophila*), whereas rGST can’t bind to these bacteria (**Figure 5E**). In addition, results from bacterial agglutination assay showed that rPcLec-EPS and rPcLec-QPS can agglutinate *S. aureus* and *V. parahaemolyticus* in the presence of Ca^2+^ (**Figure 5F**). However, BSA (as control) had no agglutination activity with tested bacteria under the same condition.

### Effects of rPcLec-EPS and rPcLec-QPS on the regulation of AMPs expression

**Figure 6:**
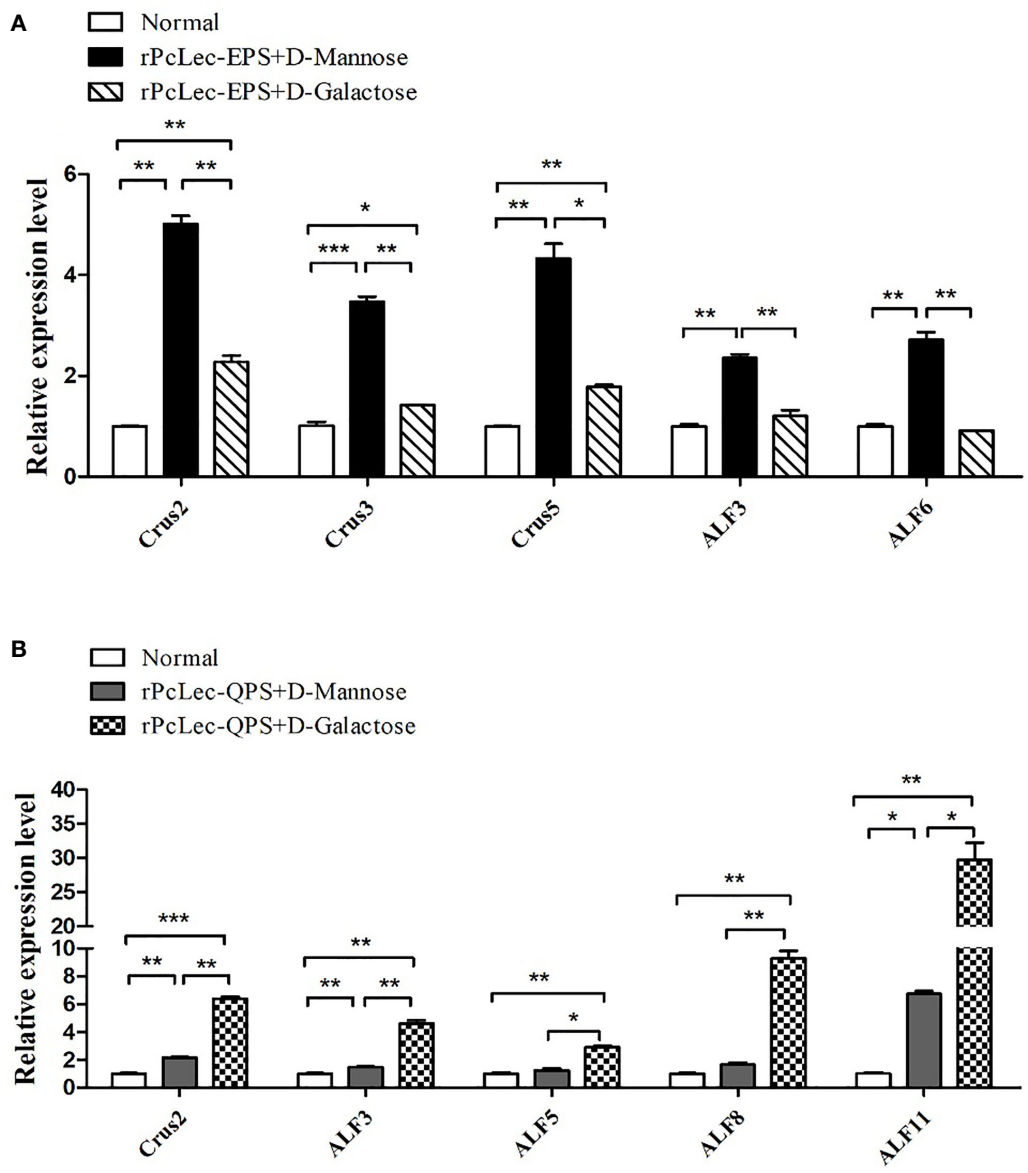
Analysis of AMPs expressions regulation by mixture of recombinant CTLs (rPcLec-EPS and rPcLec-QPS) and carbohydrates (D-Mannose and D-Galactose). **(A)** Expression levels of *Crus2*, *Crus3*, *Crus5*, *ALF3*, and *ALF6* in the gills at 24 h after injection of rPcLec-EPS and carbohydrate (D-Mannose or D-Galactose) mixture were analyzed by RT-qPCR. **(B)** Transcription levels of *Crus2*, *ALF3*, *ALF5*, *ALF8*, and *ALF11* in the gills at 24 h after injection of recombinant rPcLec-QPS and carbohydrate (D-Mannose or D-Galactose) mixture were analyzed by RT-qPCR. Each sample was composed of five crayfishes. Samples from untreated crayfishes (normal) were collected as control. Data are presented as mean ± SD, N=5. Asterisks (*p < 0.05, **p < 0.01, and ***p < 0.001) indicate that there are significant differences between two different experimental groups at the same time point.

The rPcLec-EPS and rPcLec-QPS incubated with D-Mannose or D-Galactose were injected into crayfishes to study the effects of rPcLec-EPS and rPcLec-QPS on the regulation of AMPs expression. The expression levels of *Crus2*, *Crus3*, *Crus5*, *ALF3*, and *ALF6* were increased in the rPcLec-EPS plus D-Mannose group, whereas that of *Crus2*, *Crus3*, and *Crus5* were increased in the rPcLec-EPS plus D-Galactose group compared with the normal group (**Figure 6A**). Moreover, the upregulated expressions of *Crus2*, *Crus3*, and *Crus5* in the rPcLec-EPS plus D-Mannose group were remarkably higher than that in the rPcLec-EPS plus D-Galactose group. As shown in **Figure 6B**, the expression levels of *Crus2*, *ALF3*, *ALF5*, *ALF8*, and *ALF11* were increased in the rPcLec-QPS plus D-Galactose group, whereas that of *Crus2*, *Crus3*, and *ALF11* were increased in the rPcLec-QPS plus D-Mannose group compared with the normal group. Injection of rPcLec-QPS and D-Mannose mixture made no change on the expressions of *ALF5* and *ALF8*. Furthermore, the upregulated expressions of *Crus2*, *Crus3*, and *ALF11* in the rPcLec-QPS plus D-Galactose group were remarkably higher than that in the rPcLec-QPS plus D-Mannose group. These findings indicated that injection of recombinant CTL (rPcLec-EPS or rPcLec-QPS) plus carbohydrate (D-Mannose or D-Galactose) can promote the expression of different AMPs. Moreover, combinations (rPcLec-EPS plus D-Mannose; rPcLec-QPS plus D-Galactose) were stronger to induce the expression of different AMPs.

### Effects of PcLec-EPS and PcLec-QPS knockdown on the expressions of AMPs during carbohydrate challenge

**Figure 7:**
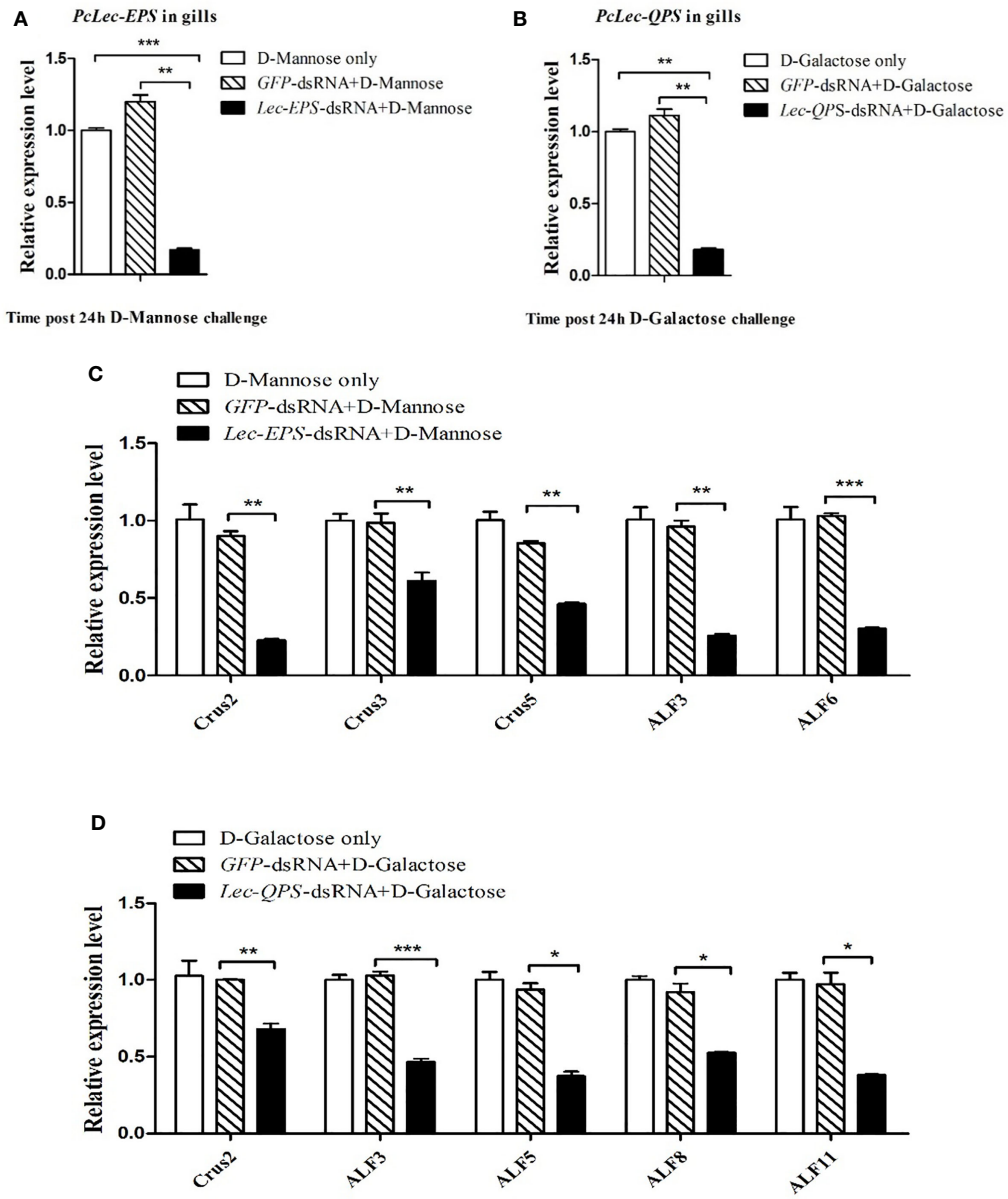
Analysis of AMPs expression regulation after carbohydrate (D-Mannose or D-Galactose) challenge in CTL (*PcLec-EPS* or *PcLec-QPS*) knockdown crayfishes. **(A)** Detection of the expression of *PcLec-EPS* at 24 h of D-Mannose challenge in the gills of *PcLec-EPS* knockdown crayfishes. **(B)** Detection of the mRNA expression of *PcLec-QPS* at 24 h of D-Galactose challenge in the gills of *PcLec-QPS* knockdown crayfishes. **(C)** Detection of the expression levels of *AMPs* (*Crus2*, *Crus3*, *Crus5*, *ALF3*, and *ALF6*) at 24 h of D-Mannose challenge in the gills of *PcLec-EPS* knockdown crayfishes. **(D)** Detection of the transcriptional levels of *AMPs* (*Crus2*, *ALF3*, *ALF5*, *ALF8*, and *ALF11*) at 24 h of D-Galactose challenge in the gills of *PcLec-QPS* knockdown crayfishes. D-Mannose only, D-Galactose only, *GFP-*dsRNA plus D-Mannose, and *GFP-*dsRNA plus D-Galactose were used as control groups. Data are presented as mean ± SD, N=5. The asterisk indicated significant difference between experimental group and control group (*p < 0.05, **p < 0.01, and ***p < 0.001).

RNAi was used to study the effects of *PcLec-EPS* and *PcLec-QPS* knockdown on the expression levels of different AMPs during D-Mannose or D-Galactose challenge. The expression level of *PcLec-EPS* in the gills of D-Mannose challenged *Lec-EPS-*dsRNA silenced crayfishes was remarkably decreased compared with that in control groups (D-Mannose only and *GFP-*dsRNA plus D-Mannose) (**Figure 7A**). The mRNA expression of *PcLec-QPS* in the gills of D-Galactose challenged *Lec-QPS-*dsRNA silenced crayfishes was remarkably decreased compared with that in control groups (D-Galactose only and *GFP-*dsRNA plus D-Galactose) (**Figure 7B**). Further studies found that knockdown of *PcLec-EPS* significantly downregulate the expressions of *Crus2*, *Crus3*, *Crus5*, *ALF3*, and *ALF6* during D-Mannose challenge (**Figure 7C**), whereas knockdown of *PcLec-QPS* obviously downregulate the expressions of *Crus2*, *ALF3*, *ALF5*, *ALF8*, and *ALF11* during D-Galactose challenge (**Figure 7D**). These results suggested that knockdown of *PcLec-EPS* inhibit the expressions of several AMPs during D-Mannose challenge, while knockdown of *PcLec-QPS* can inhibit the expressions of other AMPs when D-Galactose challenge.

### Effects of relish knockdown and injection of rPcLec-EPS or rPcLec-QPS on the expressions of AMPs

**Figure 8:**
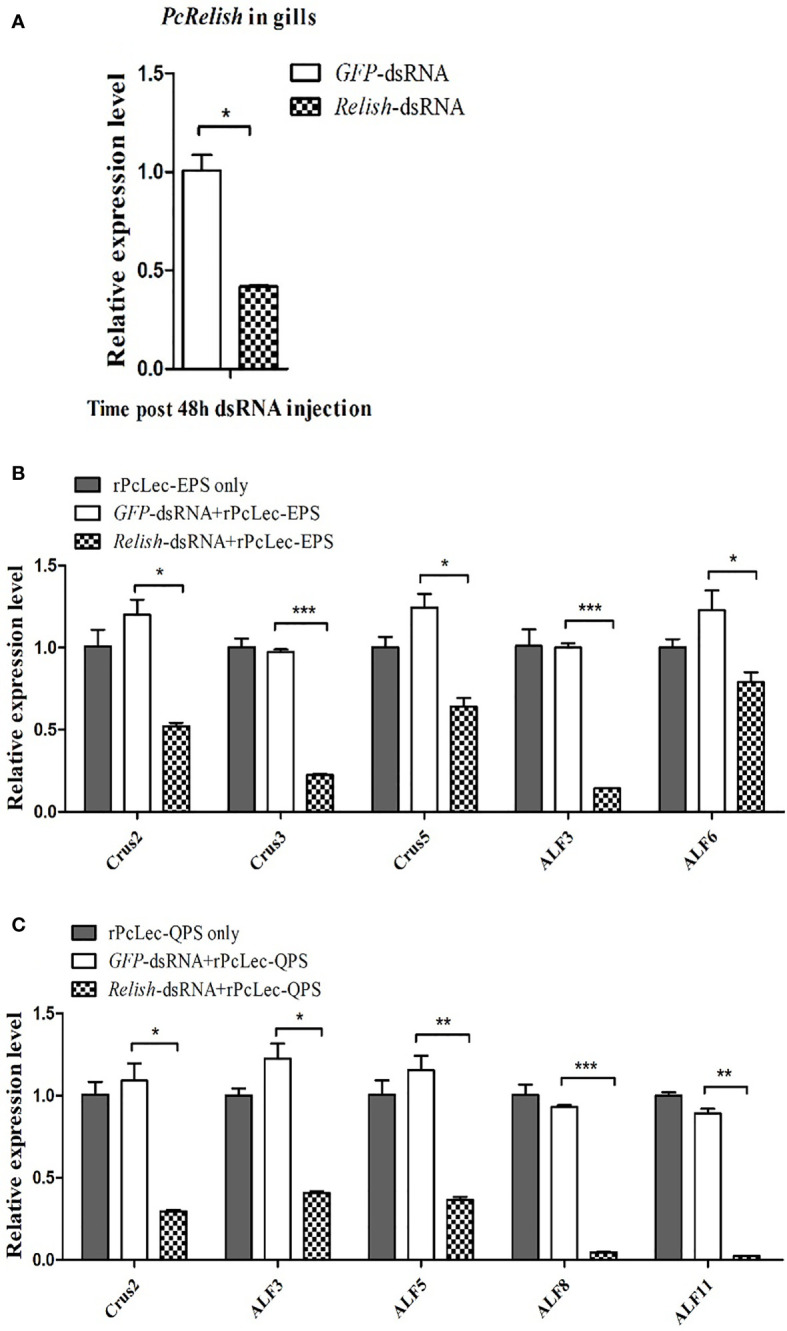
Analysis of AMPs expression regulation after recombinant CTL (rPcLec-EPS or rPcLec-QPS) injection in the *Relish* transcriptional factor knockdown crayfishes. **(A)** The expression level of *PcRelish* in the gills at 48 h after *PcRelish-*dsRNA injection was measured by RT-qPCR. Sample from *GFP-*dsRNA injected crayfishes was used as control. **(B)** Detection of the expression levels of *AMPs* (*Crus2*, *Crus3*, *Crus5*, *ALF3*, and *ALF6*) at 24 h after rPcLec-EPS injection in the gills of *Relish* knockdown crayfishes. rPcLec-EPS only and *GFP-*dsRNA plus rPcLec-EPS were used as controls. **(C)** Detection of the transcriptional levels of *AMPs* (*Crus2*, *ALF3*, *ALF5*, *ALF8*, and *ALF11*) at 24 h after rPcLec-QPS injection in the gills of *Relish* knockdown crayfishes. rPcLec-QPS only and *GFP-*dsRNA plus rPcLec-QPS were used as controls. Data are presented as mean ± SD, N=5. Asterisks indicate significant differences between experimental group and control group (*p < 0.05, **p < 0.01, and ***p < 0.001).

RNAi was used to silence *Relish* transcriptional factor in normal crayfish. As shown in **Figure 8A**, the expression level of *PcRelish* in the gills at 48 h after *Relish*-dsRNA injection was remarkably decreased compared with *GFP*-dsRNA injection group. After knockdown of *Relish*, purified rPcLec-EPS or rPcLec-QPS was injected into crayfish. At 24 h post injection, the expression levels of multiple AMPs in gills were measured by RT-qPCR. The results showed that the expression levels of *Crus2*, *Crus3*, *Crus5*, *ALF3*, and *ALF6* were significantly decreased in the *Relish*-dsRNA plus rPcLec-EPS group compared with control groups (rPcLec-EPS only and *GFP*-dsRNA plus rPcLec-EPS) (**Figure 8B**). In addition, the transcriptional levels of *Crus2*, *ALF3*, *ALF5*, *ALF8*, and *ALF11* were evidently decreased in the *Relish*-dsRNA plus rPcLec-QPS group compared with rPcLec-QPS only and *GFP*-dsRNA plus rPcLec-QPS groups (**Figure 8C**). These results reveal that the expressions of different AMPs induced by rPcLec-EPS and rPcLec-QPS depend on Relish transcriptional factor.

### Regulatory network of two CTLs, relish, and AMPs

**Figure 9:**
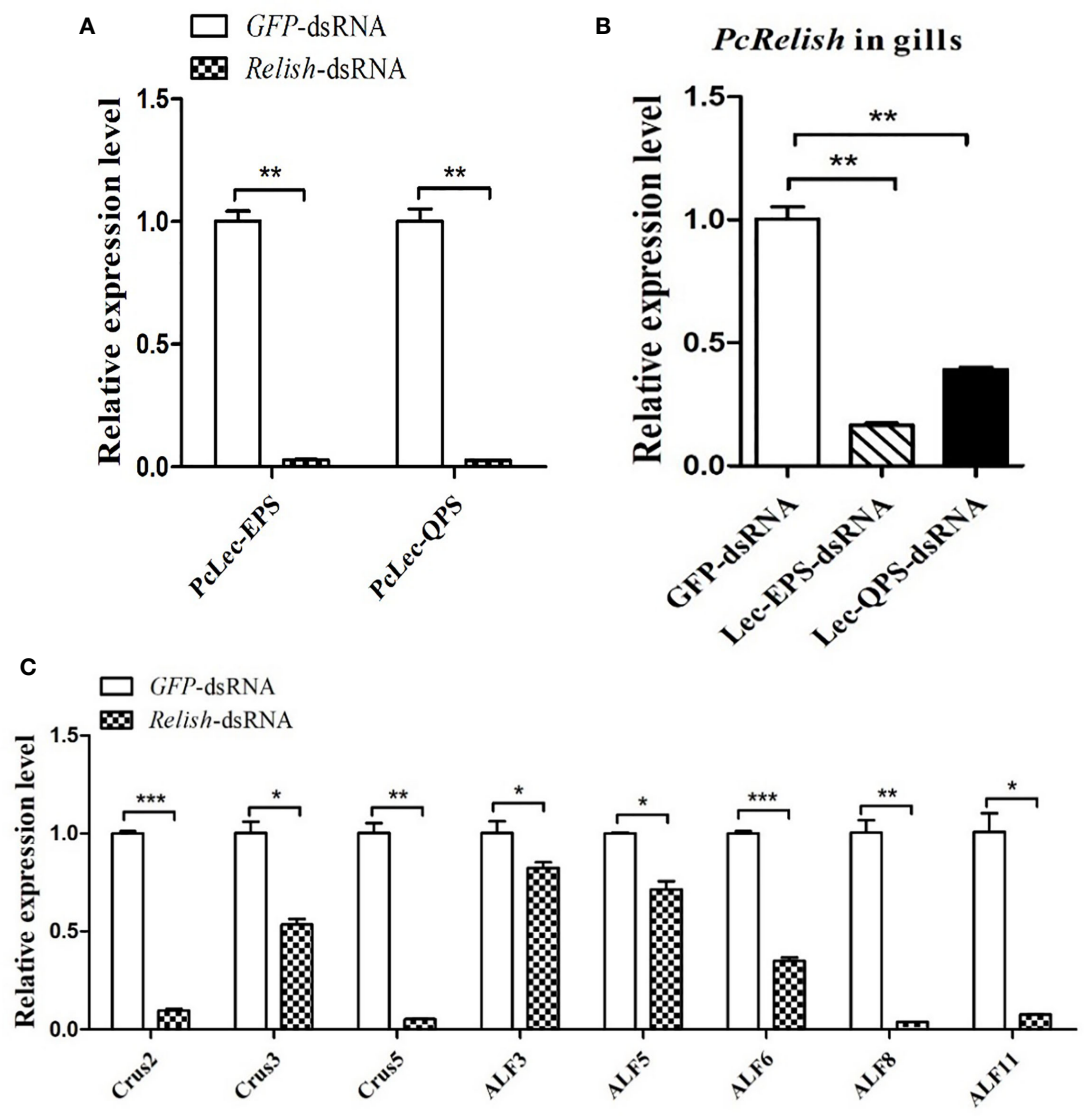
Analysis the regulatory relationship among CTLs (*PcLec-EPS* and *PcLec-QPS*), Relish, and AMPs in crayfishes. **(A)** The expression levels of *PcLec-EPS* and *PcLec-QPS* in the gills at 48 h after *Relish-*dsRNA injection were detected by RT-qPCR. **(B)** Detection of the transcriptional level of *Relish* in the gills of *Lec-EPS-*dsRNA or *Lec-QPS-*dsRNA injected crayfishes. **(C)** Detection of the expression levels of *AMPs* (*Crus2*, *3*, *5* and *ALF3*, *5*, *6*, *8*, *11*) in the gills of *Relish-*dsRNA injected crayfishes. All data were normalized to *GFP*-dsRNA treated samples. Data are presented as mean ± SD, N=5. The asterisk indicated significant difference between experimental group and control group (*p < 0.05, **p < 0.01, and ***p < 0.001).

**Figure 10:**
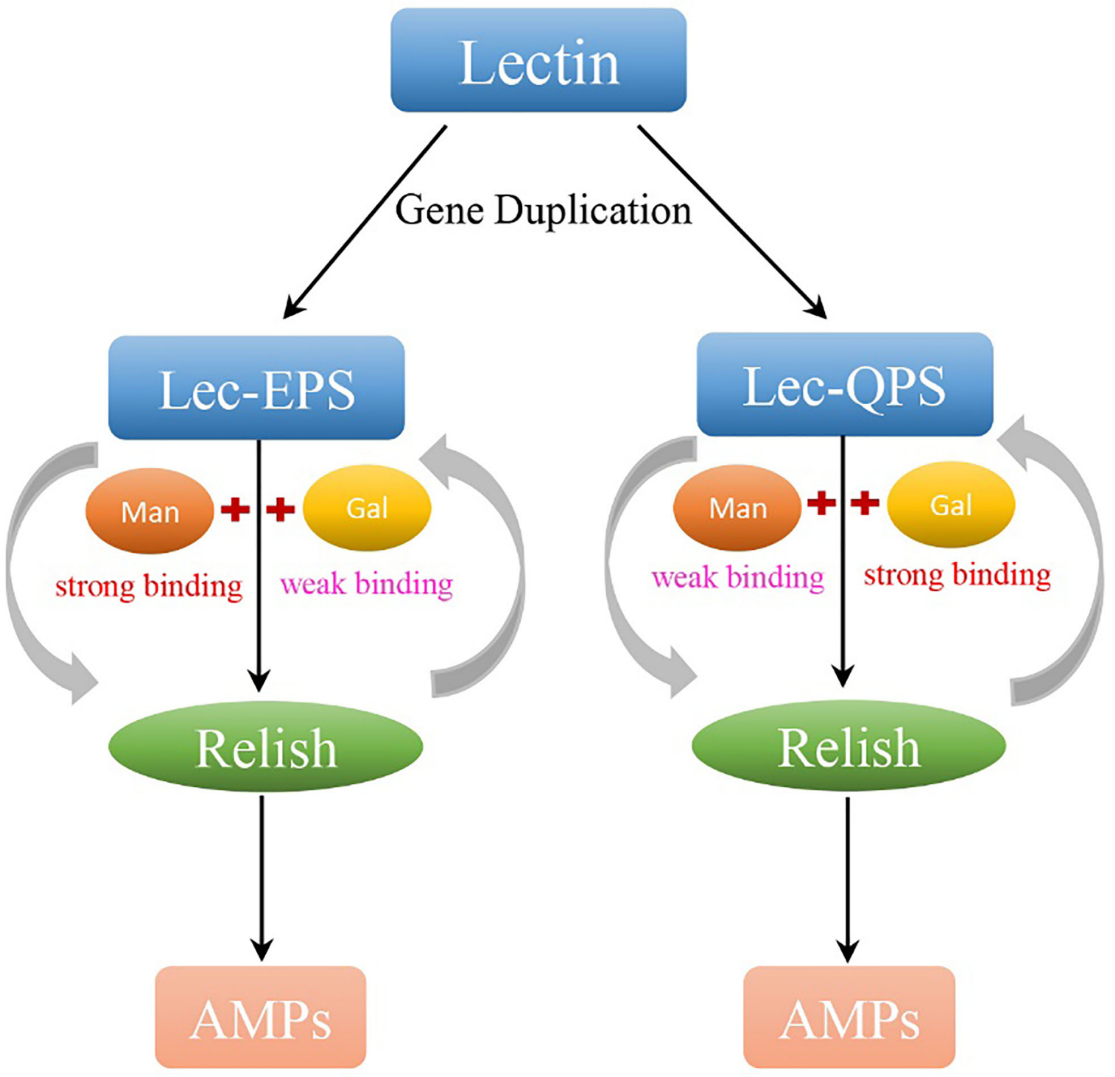
The carbohydrate recognition, immune responses, and expression regulation of PcLec-EPS and PcLec-QPS produced by GD. By GD, lectin in *P. clarkii* generates two CTLs with EPS and QPS motif, respectively. Lec-EPS and Lec-QPS in *P. clarkii* respectively strong recognize and bind D-Mannose (Man) and D-Galactose (Gal), activates the transcription of intracellular Relish, and further induces the expression of different AMPs. Moreover, the activated *Relish* positively regulates the transcription of *Lec-EPS* and *Lec-QPS*. There is a positive feedback loop between CTLs and Relish that may contribute to the expansion of the immune response for host quickly and efficiently defensing against invading microorganisms.

The regulatory relationship among two CTLs (*PcLec-EPS* and *PcLec-QPS*), Relish, and AMPs in crayfishes was explored by RNAi. As shown in **Figure 9A**, knockdown of *Relish* remarkably downregulated the expression levels of *PcLec-EPS* and *PcLec-QPS*, suggesting that *PcRelish* play a positive role in regulating the expressions of *PcLec-EPS* and *PcLec-QPS*. Moreover, knockdown of *PcLec-EPS* and *PcLec-QPS* could decrease the transcription of *PcRelish* in *P. clarkii* (**Figure 9B**), indicating that *PcLec-EPS* and *PcLec-QPS* positively regulate the expression of *PcRelish*. In addition, silence of *Relish* evidently decreased the expression levels of multiple AMPs (including *Crus2*, *3*, *5* and *ALF3*, *5*, *6*, *8*, *11*) (**Figure 9C**), suggesting that *PcRelish* positively regulate the expressions of these AMPs. Based on the above, there is a positive feedback loop between CTLs and Relish that regulates the expression of AMPs (**Figure 10**).

## Discussion

CTL genes in crustaceans undergo gene expansion. GD is a mechanism that leads to the expansion of gene families and promotes species adapting to various stressful or novel environmental conditions (26). In the present study, two novel CTLs with different carbohydrate-binding motifs from *P. clarkii* were identified and characterized. Gene cloning and sequences analysis suggested that they originate from GD. Moreover, the difference in sequences between them is mainly the exon 3 in genome. Two new-found CTLs, named as *PcLec-EPS* and *PcLec-QPS*, respectively contains EPS and QPS amino acid motif in their CRD. CTLs containing EPN and QPD motif in the CRD are thought to determine binding towards mannose and galactose, respectively (14). It’s important to note that EPN and QPD motifs always show considerable variety. EPS and QPS motifs may be variants of EPN and QPD. Hence, it’s speculated that PcLec-EPS and PcLec-QPS may have specific sugar binding activity to mannose or galactose based on the motif they bear. In previous study, we identified a hypervariable CTL gene family with different numbers of tandem repeats (Rlecs) in *Macrobrachium nipponense* that undergo gene expansion through GD and alternative splicing, which ultimately leads to functional diversity (27). The example above concluded that the expansion of the *Rlecs* family due to GD may be used by prawns to recognize different pathogens. Here, we studied the pattern recognition, immune responses, and expression regulation of *PcLec-EPS* and *PcLec-QPS* generated by GD in order to provide a clearer understanding about the significance of GD producing different CTLs and the functions of different CTLs perform in the innate immunity.

The first step of an effective immune response is immune recognition that protects hosts from invading pathogens (28). CTLs mainly rely on their CRDs to recognize and bind the conservative components of microorganisms (29). Expression patterns analysis showed that the mRNA expressions of *PcLec-EPS* and *PcLec-QPS* were remarkably increased post D-Mannose and D-Galactose challenge, suggesting that *PcLec-EPS* and *PcLec-QPS* were activated when carbohydrates challenge. Further studies found that recombinant PcLec-EPS and PcLec-QPS proteins could directly bind to carbohydrates and bacteria *in vitro*. Even more striking was rPcLec-EPS and rPcLec-QPS respectively have a higher binding activity to D-Mannose and D-Galactose, which may be caused by the EPS and QPS motifs they contain. Some other CTLs from *P. clarkii* were reported to have the capacity to bind to carbohydrates and bacteria (30–32). In addition, an important biological role of CTLs is the ability to bind carbohydrates in a calcium-dependent manner that may lead to bacterial agglutination (33). In this study, rPcLec-EPS and rPcLec-QPS could agglutinate *S. aureus* and *V. parahaemolyticus* in the presence of Ca^2+^ *in vitro*. These findings reveal that PcLec-EPS and PcLec-QPS have the characteristics of CTL family and act as PRR.

Up to now, the downstream events after recognition mediated by CTLs in invertebrates are not entirely clarified. Some studies have shown that CTLs in crustaceans are involved in the regulation of AMPs expression (34–36). In our study, knockdown of *PcLec-EPS* remarkably decreased the expression levels of *Crus2*, *Crus3*, *Crus5*, *ALF3*, and *ALF6*, whereas knockdown of *PcLec-QPS* remarkably decreased the expression levels of *Crus2*, *ALF3*, *ALF5*, *ALF8*, and *ALF11*. Moreover, injection of rPcLec-EPS plus D-Mannose mixture significantly increased the transcriptional levels of *Crus2*, *Crus3*, *Crus5*, *ALF3*, and *ALF6*, whereas injection of rPcLec-QPS plus D-Galactose significantly increased the transcriptional levels of *Crus2*, *ALF3*, *ALF5*, *ALF8*, and *ALF11*. These results indicate that *PcLec-EPS* and *PcLec-QPS* can respectively strong recognize D-Mannose and D-Galactose, and positively regulate the expressions of different crustin and anti-lipopolysaccharide factor. Results from the experiments of CTL (*PcLec-EPS* and *PcLec-QPS*) RNAi and carbohydrates (D-Mannose and D-Galactose) challenge further confirmed the above conclusion. Crustin and anti-lipopolysaccharide factor are two kinds of important AMPs with multiple groups in crustacean that have strong antimicrobial effects (37, 38). The synthetic multiple AMPs induced by *PcLec-EPS* and *PcLec-QPS* after recognizing and binding carbohydrates may further help host eliminate a variety of pathogens.

In shrimp and crayfish, PRRs-PAMPs interaction activates the NF-κB signaling pathways, such as Toll and Imd (immune deficiency) pathways, which further to regulate the expression of different sets of AMPs (13, 39). Relish transcription factor, an important part of Imd signaling pathway, plays critical roles in the production of AMPs (40). The current study found that knockdown of *Relish* in *P. clarkii* significantly inhibit the mRNA expressions of multiple AMPs activated by rPcLec-EPS or rPcLec-QPS, suggesting that *Relish* plays positive regulatory roles in the expressions of AMPs induced by PcLec-EPS or PcLec-QPS. In addition, knockdown of *PcLec-EPS* and *PcLec-QPS* could remarkably decrease the transcription of *PcRelish*, indicating that *PcLec-EPS* and *PcLec-QPS* positively regulate the expression of *Relish* transcription factor. Based on the above, there is a regulatory network of CTLs-Relish-AMPs in *P. clarkii*. Concretely, PcLec-EPS and PcLec-QPS respectively strong recognize and bind D-Mannose and D-Galactose and activates the Relish in the cytoplasm, which migrates into the nucleus to regulate the synthesis of AMPs.

In addition to study the humoral immune responses mediated by *PcLec-EPS* and *PcLec-QPS*, we also explored the regulation of expressions of these two CTLs. RNAi analysis showed that knockdown of *Relish* significantly decreased the expression levels of *PcLec-EPS* and *PcLec-QPS* in *P. clarkii*, indicating that *Relish* plays a positive regulatory role in the expressions of *PcLec-EPS* and *PcLec-QPS*. As NF-κB transcription factor, study has shown that Dorsal in *Litopenaeus vannamei* can activate the promoter of LvCTL3 (22). And, the promoter of LvCTL4 can be activated by Relish transcription factor (23). Furthermore, the activating capacity of Relish is higher than that of Dorsal. These reports suggest sthat the expression levels of CTLs can be regulated by Dorsal and Relish transcription factor. However, whether the expressions of *PcLec-EPS* and *PcLec-QPS* are regulated by Dorsal needs more research. In addition, knockdown of the expression of *Relish* leads to the decreased expressions of *Crus* (*2*, *3*, and *5*) and *ALF* (*3*, *5*, *6*, *8*, and *11*). These findings reveal that there is a positive feedback loop between CTLs (*Lec-EPS* and *Lec-QPS*) and Relish that regulates the expression of AMPs.

In conclusion, two CTLs with EPS and QPS motif respectively (named *PcLec-EPS* and *PcLec-QPS*) were identified from *P. clarkii*. *PcLec-EPS* and *PcLec-QPS* were produced by GD. Carbohydrates (D-Mannose and D-Galactose) challenge activated the expressions of *PcLec-EPS* and *PcLec-QPS*. Studies *in vitro* showed that recombinant PcLec-EPS and PcLec-QPS protein can bind to carbohydrates and microbes, and agglutinate bacteria. Moreover, rPcLec-EPS and rPcLec-QPS showed a stronger binding activity to D-Mannose and D-Galactose, respectively. Studies *in vivo* showed that the strong recognition and binding of PcLec-EPS and PcLec-QPS to D-Mannose and D-Galactose leads to the activation of Relish transcriptional factor, and upregulation of different AMP expression. In addition, two CTLs and Relish could positively regulate the expression of each other. Therefore, there is a positive feedback loop between CTLs (PcLec-EPS and PcLec-QPS) and Relish that regulates the expression of AMPs, which may contribute to the expansion of the immune response for host quickly and efficiently eliminating pathogenic microorganisms.

## Data availability statement

Publicly available datasets were analyzed in this study. This data can be found on Genbank: OP450963, and Procambarus clarkii Lec-EPS mRNA, complete cds - Nucleotide - NCBI (nih.gov): OP450964.

## Ethics statement

Ethical review and approval were not required for the animal study because the research species of this manuscript is a lower invertebrate – crayfish (*Procambarus clarkii*), which does not require ethical certification.

## Author contributions

XD, funding acquisition, data curation, formal analysis, investigation, methodology, software, supervision, validation, visualization, and writing - original draft. MS, funding acquisition, data curation, formal analysis, investigation, methodology, supervision, validation, and visualization. XN, data curation, formal analysis, methodology, software, and visualization. YZ, data curation, formal analysis, software, and validation. HX, formal analysis, methodology, and validation. ZH, software and visualization. TG, project administration, funding acquisition, and resources. XH, project administration, funding acquisition, resources, and writing - review and editing. QR, conceptualization, project administration, resources, and writing - review and editing. All authors contributed to the article and approved the submitted version.

## Funding

The current study was supported by the National Natural Science Foundation of China (Grant Nos. 31902397), the Natural Science Foundation of Jiangsu Province (BK20190698), the “JBGS” Project of Seed Industry Revitalization in Jiangsu Province (No. JBGS〔2021〕031), the National Key R&D Program of China (2018YFD0901303), the Agricultural Project from Jiangsu Province Science and Technology Agency (no. BE2020348), the Jiangsu Agricultural Industry Technology System (no. JATS [2022] 344 and no. JAST [2022] 412), and the Postgraduate Research & Practice Innovation Program of Jiangsu Province (1812000024880).

## Conflict of interest

The authors declare that the research was conducted in the absence of any commercial or financial relationships that could be construed as a potential conflict of interest.

## Publisher’s note

All claims expressed in this article are solely those of the authors and do not necessarily represent those of their affiliated organizations, or those of the publisher, the editors and the reviewers. Any product that may be evaluated in this article, or claim that may be made by its manufacturer, is not guaranteed or endorsed by the publisher.
